# Comment on “The power metric: a new statistically robust enrichment-type metric for virtual screening applications with early recovery capability”

**DOI:** 10.1186/s13321-018-0267-x

**Published:** 2018-03-15

**Authors:** M. Šícho, M. Voršilák, D. Svozil

**Affiliations:** 1 CZ-OPENSCREEN:National Infrastructure for Chemical Biology, Department of Informatics and Chemistry, Faculty of Chemical Technology, University of Chemistry and Technology Prague, Prague, Czech Republic; 2 CZ-OPENSCREEN: National Infrastructure for Chemical Biology, Institute of Molecular Genetics, AS CR v.v.i, Prague, Czech Republic

Recently, a new metric for virtual screening applications was reported by Lopes et al. [1]. This metric is called the power metric (*PM*) as it is based on the principles of the statistical power of a hypothesis test. In this comment, we add to the original article and discuss the similarity of *PM* to precision (*Pre*) and draw new conclusions from their functional relationship.

*PM* is defined as:

1 $$PM = \frac{TPR}{TPR + FPR}$$

and can be reformulated as follows:

2 $$PM = \frac{TPR}{TPR + FPR} = \frac{{\frac{TP}{TP + FN}}}{{\frac{TP}{TP + FN} + \frac{FP}{FP + TN}}} = \frac{{\frac{TP}{P}}}{{\frac{TP}{P} + \frac{FP}{N}}} = \frac{N \cdot TP}{N \cdot TP + P \cdot FP} = \frac{{\frac{N \cdot TP}{N}}}{{\frac{N \cdot TP + P \cdot FP}{N}}} = \frac{TP}{{TP + \frac{P}{N}FP}}$$

In this formula, *P* is a total number of positive and *N* a total number of negative examples in a data set. Similarly, *Pre* is defined as:

3 $$Pre = \frac{TP}{TP + FP} = \frac{TPR}{{TPR + \frac{N}{P}FPR}}$$

From the comparison of Eqs. 2 and 3 follows that *PM* differs from *Pre* by the $\frac{P}{N}$ term which precedes the number of false positives *FP* in *PM*. Thus, the influence of *FP* in *PM* is decreased in imbalanced data sets with a high number of negative examples and the magnitude of this effect directly depends on the $\frac{P}{N}$ ratio. Due to this dependency, *PM* has the ability to cancel out the influence of negative examples and is, in this regard, more robust than *Pre*.

*Pre* and *PM* are, however, not mutually exclusive and depend on each other. From Eqs. 1 and 3, the following functional relationship can be derived:

4 $$\frac{PM}{Pre} = \frac{{\frac{TPR}{TPR + FPR}}}{{\frac{TPR}{{TPR + \frac{N}{P}FPR}}}} = \frac{{TPR + \frac{N}{P}FPR}}{TPR + FPR} = \frac{{TPR + FPR - FPR + \frac{N}{P}FPR}}{TPR + FPR} = 1 + \frac{{\left( {\frac{N}{P} - 1} \right)FPR}}{TPR + FPR}$$

Because of this relationship, both *PM* and *Pre* capture model performance trends in a very similar way as we will demonstrate further.

**Fig. 1:**
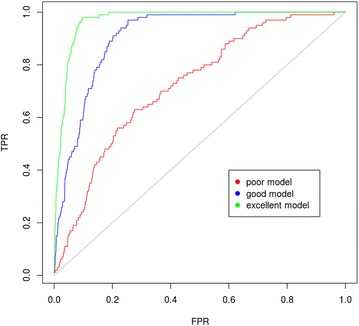
ROC curves of three generated models. A P/N of each model was set to 100/9900. The poor quality model was obtained by setting a *λ* parameter to 3, the good quality model has a *λ* parameter equaling to 10 and *λ* in the excellent quality model was set to 30. A diagonal gray line shows the ROC of a random model

**Fig. 2:**
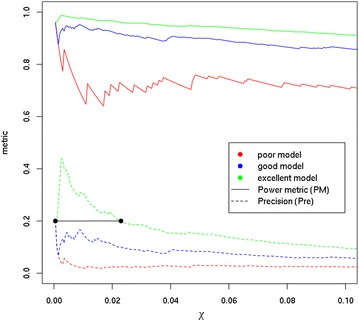
The influence of *χ* cutoff on the shape of both *PM* and *Pre* metrics for models of various quality. Y-axis shows the values of *PM* (full line) or *Pre* (dashed line) metrics. The black line segment delimits a *χ* range in which a small change in *χ* leads to a significant change in *Pre* for good and excellent models, i.e., to the acceptance of a large number of false positives

Using the same approach as described in [1], we generated three models with $\frac{P}{N} = \frac{100}{9900}$: one of poor quality (*λ *= 3), one of good quality (*λ *= 10) and one of excellent quality (*λ *= 30) (Fig. 1). Each model yields an ordered set of compounds from which a fraction of molecules, defined by the cutoff threshold *χ*, is selected as hits (i.e., *FP* + *TP*). The influence of *χ* cutoff on both metrics in the early recovery region with *χ *< 0.1 is shown in Fig. 2.

Figure 2 clearly shows that both *PM* and *Pre* capture the same trends, albeit at different scales. For a poor quality model, *PM* values vary considerably more than *Pre* values, which is due to the $\frac{P}{N}$ ratio. While *PM* is more sensitive to the increase in accepted actives ($\frac{P}{N}$ decreases the influence of false positives for *PM*, see Eq. 2), *Pre* value shows less variance and it quickly approaches zero because the list of the top hits gets “flooded” with false positives. On the other hand, for good and excellent quality models we find more variance in *Pre* than in *PM* (Fig. 2). In particular for an excellent quality model, *PM* varies very little, again due to the influence of $\frac{P}{N}$. Therefore using *Pre*, one can identify a range of *χ* values where a small shift in *χ* results in the acceptance of a large number of false positives (Fig. 2, black line segment). This effect is, however, not captured so distinctively by *PM*.

Therefore, we may conclude that the main advantage of *PM* over *Pre* is its robustness with respect to the imbalance of positive and negative examples. However, *PM* fails to capture, especially for well-performing models, the influence of false positives. In addition, *PM* and *Pre* metrics are in a functional relationship. Therefore, if *PM* and *Pre* are used for the comparison of two different models on the same data set, the conclusions are the same irrespective of the metric. Lastly, it is also important to note that when the $\frac{P}{N}$ ratio equals to 1 (i.e., in a balanced data set), *PM* and *Pre* become equivalent.

In the end, we would like to emphasize that *PM* is not a suitable metric for the performance assessment of classification models. Similarly to *Pre*, it does not take into account the number of true or false negatives. Thus, it should be accompanied by a metric taking negative classifications into account, just as *Pre* is commonly reported together with a recall.
